# Anti-Atherogenic Activity of Polyphenol-Rich Extract from Bee Pollen

**DOI:** 10.3390/nu9121369

**Published:** 2017-12-18

**Authors:** Anna Rzepecka-Stojko, Jerzy Stojko, Krzysztof Jasik, Ewa Buszman

**Affiliations:** 1 Department of Pharmaceutical Chemistry, School of Pharmacy with the Division of Laboratory Medicine in Sosnowiec, Medical University of Silesia in Katowice, Jagiellońska 4, 41-200 Sosnowiec, Poland; ebuszman@sum.edu.pl; 2 Department of Toxicology and Bioanalysis, School of Pharmacy with the Division of Laboratory Medicine in Sosnowiec, Medical University of Silesia in Katowice, Jagiellońska 4, 41-200 Sosnowiec, Poland; jstojko@sum.edu.pl; 3 Department of Skin Structural Studies, School of Pharmacy with the Division of Laboratory Medicine in Sosnowiec, Medical University of Silesia in Katowice, Kasztanowa 3, 41-200 Sosnowiec, Poland; kjasik@sum.edu.pl

**Keywords:** bee pollen, polyphenols, atherosclerosis, oxidised low density lipoproteins (ox-LDL), asymmetric dimethylarginine (ADMA), angiotensin-converting enzyme (ACE), angiotensin II (ANG II)

## Abstract

The aim of this study was to determine the effect of polyphenol-rich ethanol extract of bee pollen (EEP) on atherosclerosis induced by a high-fat diet in ApoE-knockout mice. EEP was given with feed in two doses of 0.1 and 1 g/kg body mass (BM). The studies have been conducted in a period of 16 weeks. The following factors were estimated: total cholesterol (TC), oxidized low density lipoproteins (ox-LDL), asymmetric dimethylarginine (ADMA), angiotensin-converting enzyme (ACE) and angiotensin II (ANG II) in the 5th, 10th, 12th, 14th, and 16th week of the experiment. In the last, i.e., 16th week of the studies the development of coronary artery disease (CAD) was also estimated histopathologically. Supplementing diet with EEP resulted in decreasing TC level. EEP reduced oxidative stress by lowering the levels of ox-LDL, ADMA, ANG II and ACE. EEP protected coronary arteries by significantly limiting the development of atherosclerosis (the dose of 0.1 g/kg BM) or completely preventing its occurrence (the dose of 1 g/kg BM). The obtained results demonstrate that EEP may be useful as a potential anti-atherogenic agent.

## 1. Introduction

Recently, a lot of studies have focused on pro-health and curative properties of natural products, including bee products, and bee pollen is among these valuable apitherapeutics. It is a product of plant origin, collected and partly processed by bees. It is produced from pollen grains enriched with nectar, honey and honeybee salivary glands secretion. Bee pollen is composed of more than 250 various substances. The type and role of separate compounds in the total mass is diverse, and depend on plant species, the climate zone and season when it was collected [1,2,3].

Bee pollen is characterized by high nutritious value and different biological activities. Nutritious properties of bee pollen result from the presence of such substances as proteins, amino acids, carbohydrates, lipids (including ω-3 and ω-6 acids), vitamins and bio-elements. Therapeutic and protective effects are related to the content of functional compounds such as polyphenols [4,5,6,7].

Polyphenols are the main ingredients that determine antioxidant activity of bee pollen [8,9]. Their content may vary significantly depending on the origin of the material [10,11,12]. The profile of polyphenolic compounds in bee pollen may serve as an important indicator of the product quality due to its specificity as well as qualitative and quantitative stability [13,14]. Polyphenols in bee pollen are mainly phenolic acids and flavonoids, and their biological activity is conditioned by their specific chemical structure. The presence of a benzene ring in the particle structure is a common characteristic of the compounds. High antioxidant capacity of polyphenols is closely related to the presence of conjugated double bonds and the number and location of hydroxyl groups in an aromatic ring [15,16]. Since polyphenols can neutralize free radicals, they have the following activities: anti-inflammatory, antiallergenic, antiviral, anti-clotting, anticancer, immunostimulating, hepatoprotective, and they also inhibit specific enzymes. Furthermore, polyphenols have a protective function in cardio-vascular diseases caused by oxidative stress [17,18,19].

Cardio-vascular diseases, including cardiac artery disease, are a frequent cause of death in the majority of highly developed countries. Atherosclerosis is a specific form of a chronic inflammatory process in the aorta and medium-sized arteries. Pathogenesis of atherosclerosis is caused by various factors. The development of atherosclerosis is modulated by disturbances of lipid metabolism, oxidative stress, fibrinolysis and coagulation in the vascular wall as well as disturbed homeostasis of the renin-angiotensin-aldosterone system, which impairs the function of endothelial cells [20,21].

Taking into consideration various biological activities of bee pollen ingredients, the aim of the current study was to determine the effect of polyphenol-rich EEP on atherosclerosis induced by a high-fat diet in ApoE-knockout mice. The latter constitute an internationally recognized animal model for studying atherosclerosis and hypercholesterolemia.

## 2. Materials and Methods

### 2.1. Reagents and Drugs

Xylene, hematoxylin, eosin aqueous solution (1%), anhydrous ethyl alcohol (99.8%), acetone, phosphate buffered saline (PBS), formalin were purchased from POCh (Gliwice, Poland). DPX mounting medium for histology (mixture of Distyrene, a Plasticizer, and Xylene) was purchased from Fluka (Dresden, Germany). Tiopental was purchased from Sandoz (Warsaw, Poland), total cholesterol Test (Cat. No. OSR 6116) was purchased from Beckman Coulter (Praha, Czech Republic), ELISA Kit for mouse oxidized low density lipoprotein (Cat. No. E90527Mu) was purchased from Uscn Life Science Inc. (Wuhan, China), ADMA ELISA Kit for the determination of ADMA (Cat. No. K 3001) was purchased from Immundiagnostik AG (Bensheim, Germany), ELISA Kit for mouse angiotensin I converting enzyme (ACE) (Cat. No. E90004Mu) was purchased from Uscn Life Science Inc. (Wuhan, China), Angiotension II (Human, Rat, Mouse, Porcine, Caniane) EIA Kit (Cat. No. EK-002-12) was purchased from Phoenix Pharmaceutical Inc. (Karlsruhe, Germany).

### 2.2. Preparation of Ethanol Extract of Bee Pollen (EEP)

The material for the tests were ground pollen loads obtained from ecologically clean harvest areas in the south of Poland. Samples of polyfloral bee pollen were collected from an apiary at Kamianna (GPS N 49°31′527, E 20°56′116), Poland, in the Beskidy Mountains. From May to July, bee pollen samples were collected by beekeepers with the use of pollen traps mounted on selected beehives. Mainly, they were pollens of common dandelion *Taraxacum officinale*, rapeseed *Brassica napus*, European raspberry *Rubus ideaeus*, acacia *Robinia pseudoacacia*, buckwheat *Fagopyrum esculentum*, linden *Tilia mordata*, clover *Trifolium repens*. Ethanol extract was prepared according to a slightly modified method of Almaraz-Abarca et al. [8]. The ethanol extract of bee pollen was prepared by weighing 20 g of ground bee pollen, with accuracy of 0.01 g. Then, the bee pollen sample was extracted 5 times with 50% (*v*/*v*) ethanol aqueous solution, in 200 mL portions, and shaken each time for 60 min at room temperature, in order to macerate the sample. After each extraction, the sample was filtered under reduced pressure with the use of a water pump. The filtrate was collected, and substrate was extracted again with another portion of ethanol aqueous solution. The obtained filtrate was centrifuged at 10,000 rpm for 10 min, and then it was evaporated under reduced pressure in a rotary vacuum evaporator (UNIPAN-PRO 350P). The evaporated extract was dried in a laboratory incubator at 38 °C to obtain solid mass. Studies of EEP focused on the content of polyphenols and flavonoids, and their antioxidant effect [22].

### 2.3. Animals and Treatments

The study was conducted on 56 females of C_57_BL_6_ ApoE-knockout mice, aged 4 weeks, 25 ± 5 g of body mass. During the experiment, the animals were kept in standard breeding conditions, i.e., in groups of 10 animals in polypropylene cages, in rooms of constant temperature (23 ± 2 °C), and constant air humidity (50–70%), keeping the daytime rhythm in the inflow of light (a 12-h cycle). The high-fat diet (HFD) comprised High Fat Rodent Diet supplemented with 21% lard and 0.15% cholesterol (Special Diets Services, Witham, Essex, UK). The standard diet (SD) was standard feed (Labofeed B) containing 17% protein, 3.5% fat and 38% carbohydrates (Animal Feed Manufacturer “Morawski” in Kcynia). EEP was added to the feed in a dose of 0.1 and 1 g/kg BM, respectively. Two dosing levels of EEP used in the research were calculated based on the daily ingestion of polyphenols in the diet of the inhabitants of Central Europe [23].

Experimental animals were divided into 6 groups according to the following scheme: 5 study groups: HFD, HFD-0.1, HFD-1, SD-0.1, SD-1 (10 animals in each group), and a control group SD (6 animals). Characteristics of the groups: HFD: high-fat diet, HFD-0.1: high-fat diet supplemented with EEP (0.1 g/kg BM), HFD-1: high-fat diet supplemented with EEP (1 g/kg BM), SD-0.1: standard diet supplemented with EEP (0.1 g/kg BM), SD-1: standard diet supplemented with EEP (1 g/kg BM), and SD: standard diet.

The project with the use of animal models was approved by the Local Ethics Committee on Animal Experimentation of the Medical University of Silesia in Katowice.

### 2.4. Collecting Biological Material for Tests

The material for tests was collected in the fasted state, in the 5th, 10th, 12th, 14th, and 16th week of the experiment, after general anesthesia with a drug called Tiopental in a dose of 20 mg/kg BM, administered by injection.

Blood for analyses was collected by puncturing the tip of the heart with a cannula. Blood was collected to chemically pure test tubes in order to obtain serum. Blood after clotting and clot retraction was centrifuged, and serum was frozen at −80 °C, and stored for further tests.

The collection of material for histopathological tests was as follows: before the material was collected, perfusion was performed by decompression surgery in the 1/3 of descending abdominal aorta. Perfusion was performed with phosphate buffered saline (PBS) until the light pink translucent liquid flew out in the decompressing orifice. Then, perfusion with 10% PBS-buffered formaldehyde was performed for 2 min to obtain preliminary fixing of the heart and trunks of major blood vessels. Next, both, the heart and the brachiocephalic trunk were fixed in 10% PBS-buffered formaldehyde, and kept for further tests.

### 2.5. Histopathological Tests

Histopathological evaluation of the heart and the brachiocephalic trunk was conducted in the 16th week of the study. The collected material was fixed in a 10% formalin solution for minimum 24 h. After fixing, the tissues were treated with the aqueous solutions of ethyl alcohol. The material was subsequently rinsed with acetone and xylene, respectively. Then, tissues were placed in a xylene and paraffin mixture at a 1:1 ratio and then in liquid paraffin. After solidification, the paraffin was cut into 3–5 micron thick bands. Paraffin bands were treated with xylene, an ethyl alcohol/xylene mixture at a 1:1 ratio and aqueous solutions of ethyl alcohol, and then rinsed in distilled water. The material prepared in this way was stained with the standard hematoxylin-eosin (HE) method, which included staining with alkaline solution of hematoxylin, rinsing in distilled water, staining with acid solution of eosin, and rinsing in distilled water. Later, preparations were treated with aqueous solutions of ethyl alcohol, ethanol/xylene mixture at 1:1 ratio and rinsed in xylene in order to finally dehydrate and radiate the tissue. Slides were covered with a cover glass with DPX-medium.

Histopathological preparations were evaluated in 40×, 100×, and 200× microscopy objectives magnification using Olympus BX60 microscope equipped with XC50 digital camera and Olympus cellSens Standard software (Olympus Corp., Tokyo, Japan).

### 2.6. Biochemical Tests

We tested serum obtained in the scheduled study periods, i.e., in the 5th, 10th, 12th, 14th, and 16th week of the experiment for the study groups HFD, HFD-0.1, HFD-1, SD-0.1, SD-1, and in the 1st, 10th, and 16th week for the control group (SD). The levels of ox-LDL, ADMA, ACE, ANG II were determined with Asys HiTech UVM 340 microplate reader and Micro Win 4.35.

#### 2.6.1. Determination of Total Cholesterol (TC) 

Total cholesterol serum level was determined with an enzymatic method. A reagent set from Beckman Coulter (Cat. No. OSR 6116) was used for the determination, which was conducted according to the manufacturer’s instruction for Beckman Coulter AU 680 analyzer. Total cholesterol level was given in mg/dL.

#### 2.6.2. Determination of Oxidized Low Density Lipoprotein (Ox-LDL) Concentration

The concentration of ox-LDL was determined with a sandwich ELISA using Mouse Oxidized Low Density Lipoprotein ELISA Kit. The analysis was conducted according to the manufacturer’s instruction.

The microtiter plate was pre-coated with an antibody specific to ox-LDL. Samples diluted 10 times or standards were then added to the appropriate microtiter plate wells, and incubated for 2 h (37 °C). Then, the liquid was removed and a biotin-conjugated polyclonal antibody specific for ox-LDL was added. The plate was incubated for 1 h at 37 °C. Then the plate was washed, and avidin conjugated to horseradish peroxidase (HRP) was added to each microplate well, and the plate was incubated for 1 h (37 °C). Next, a 3,3′,5,5′-tetramethyl-benzidine (TMB) substrate solution was added to each well and incubated for 30 min (37 °C). Only those wells that contained ox-LDL, biotin-conjugated antibody and enzyme-conjugated avidin exhibited a change in color. The enzyme-substrate reaction was terminated by the addition of a sulfuric acid solution, and the color change was measured spectrophotometrically at a wavelength of 450 nm. The concentration of ox-LDL (ng/mL) in the samples was then determined by comparing the optical density (O.D.) of the samples to the standard curve.

#### 2.6.3. Determination of Asymmetric Dimethylarginine (ADMA) Concentration

The concentration of ADMA was determined with ADMA ELISA Kit for the determination of ADMA. The assay was conducted according to the manufacture’s instruction.

This assay is based on the method of competitive enzyme-linked immunoassays. The sample preparation includes the addition of a derivatization-reagent for ADMA coupling. Afterwards, the treated samples and the polyclonal ADMA-antiserum are incubated in wells of microplate coated with ADMA-derivative (tracer) for 18 h at 4–8 °C. During the incubation period, the target ADMA in the sample competes with the tracer immobilized on the wall of the microtiter wells for the binding of the polyclonal antibodies. The ADMA in the sample displaces the antibodies out of the binding to the tracer. Therefore the concentration of the tracer-bound antibody is inversely proportional to the ADMA concentration in the sample. During the second incubation step (for 1 h at 18–26 °C), a peroxidase-conjugated antibody is added to each microtiter well to detect the anti-ADMA antibodies. After washing away the unbound components, 3,3′,5,5′-tetramethyl-benzidine (TMB) is added as a substrate for peroxidase and then incubated for 10 min at 18–26 °C. Finally, the enzymatic reaction is terminated by acidic stop solution. The color changes from blue to yellow and the absorbance is measured in the photometer at 450 nm. The intensity of the yellow color is inversely proportional to the ADMA concentration in the sample; this means high ADMA concentration in the sample reduces the concentration of tracer-bound antibodies and lowers the photometric signal. The concentration of ADMA (μmol/L) in the samples is then determined by comparing the O.D. of the samples to the standard curve. 

#### 2.6.4. Determination of Angiotensin-Converting Enzyme (ACE) Concentration

The concentration of ACE was determined with a sandwich ELISA using mouse angiotensin I converting enzyme (ACE) ELISA Kit. The assay was conducted according to the manufacturer’s instruction.

The microtiter plate in this kit was pre-coated with an antibody specific to ACE. Samples diluted 10 times or standards were added to the appropriate microtiter plate wells and incubated for 2 h (37 °C). Then the liquid was removed and a biotin-conjugated polyclonal antibody specific for ACE was added. The plate was incubated for 1 h at 37 °C. Then the plate was washed, and avidin conjugated to horseradish peroxidase (HRP) was added to each microplate well, and incubated for 1 h (37 °C). Then a 3,3′,5,5′-tetramethyl-benzidine (TMB) substrate solution was added to each well and incubated for 15 min (37 °C). Only those wells that contained ACE, biotin-conjugated antibody and enzyme-conjugated avidin would exhibit a change in color. The enzyme-substrate reaction was terminated by the addition of a sulphuric acid solution and the color change was measured spectrophotometrically at a wavelength of 450 nm. The concentration of ACE (ng/mL) in the samples was then determined by comparing the O.D. of the samples to the standard curve. 

#### 2.6.5. Determination of Angiotensin II (ANG II) Concentration

The concentration of ANG II was determined with Angiotension II (Human, Rat, Mouse, Porcine, Caniane) EIA Kit. The assay was carried out according to the manufacturer’s instruction. 

The microtiter plate in this kit was pre-coated with an antibody specific to ANG II. Samples diluted 10 times or standards were then added to the appropriate microtiter plate wells with a biotin-conjugated polyclonal antibody preparation specific for ANG II and incubated for 2 h (20–23 °C). Then streptavidin conjugated to Horseradish Peroxidase (SA-HRP) was added to each microplate well and incubated 1 for hour (20–23 °C). Next a 3,3′,5,5′-tetramethyl-benzidine (TMB) substrate solution was added to each well and incubated for 1 h (20–23 °C). Only those wells that contained ANG II, biotin-conjugated antibody and enzyme-conjugated streptavidin would exhibit a colour change. The enzyme-substrate reaction was terminated by the addition of a hydrochloric acid solution, and the colour change was measured spectrophotometrically at a wavelength of 450 nm. The concentration of ANG II (ng/mL) in the samples was then determined by comparing the O.D. of the samples to the standard curve. 

### 2.7. Statistical Analysis

The obtained results in particular groups were given as the mean ± standard deviation (±SD), and checked for normal distribution and homogeneity of these groups. The Shapiro-Wilk test was performed for normality of the obtained results. Homogeneity of variance was evaluated with the Levene’s test with two variables. The comparison of homogenous groups and the effect of parameters (mouse model, diet type, supplement dose) on group differentiation were analyzed with ANOVA and the Least Significant Differences (LSD) test.

The differences were considered to be statistically significant when a significance level was less than 0.05 (*p* ≤ 0.05). The calculations were performed with Statistica 10.0 (Polish version), and Microsoft Excel.

## 3. Results and Discussion

### 3.1. Histopathological Tests of Arteries

**Figure 1:**
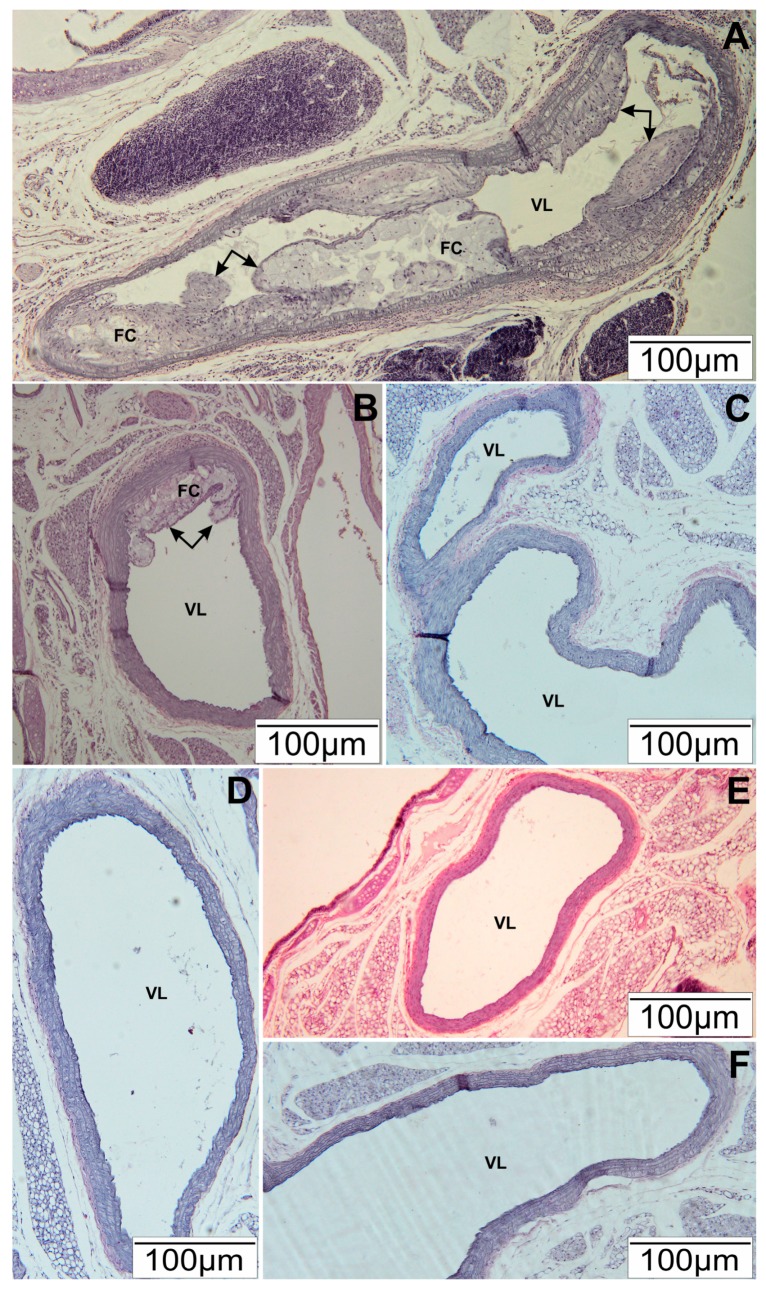
Representative section of the aortic arch of ApoE-knockout mice. Slides stained Hematoxylin and Eosin. (**A**) HFD—high-fat diet. Arrows—atherosclerotic plaque, FC—foam cells, VL—vascular lumen; (**B**) HFD-0.1—high-fat diet supplemented with EEP 0.1 g/kg BM. Arrows—atherosclerotic plaque, FC—foam cells, VL—vascular lumen; (**C**) HFD-1—high-fat diet supplemented with EEP 1 g/kg BM. VL—vascular lumen; (**D**) SD-0.1—standard diet supplemented with EEP 0.1 g/kg BM. VL—vascular lumen; (**E**) SD-1—standard diet supplemented with EEP 1 g/kg BM; (**F**) SD—standard diet. VL—vascular lumen. Scale Bar: 100 µm.

In our study, we evaluated the anti-atherogenic effect of polyphenol-rich extract of bee pollen on the development of atherosclerosis induced by a high-fat diet in ApoE-knockout mice. We showed that supplementation of a high-fat diet with EEP in a dose of 1 g/kg BM protected heart arteries from development of atherosclerotic plaque. Histopathological presentation did not reveal any proliferative changes which would be evidence of atherosclerosis development (Figure 1C). Supplementing a high-fat diet with EEP in a dose of 0.1 g/kg BM significantly limited the growth of atherosclerotic plaque (Figure 1B), while severe atherosclerotic changes occurred in mice on a high-fat diet without supplementation. Near the aortic arch, atherosclerotic plaque almost completely filled the vascular lumen (Figure 1A). In mice on a standard diet, the control group SD (Figure 1F), and in the mice on a standard diet supplemented with EEP in a dose of 0.1 g/kg BM (Figure 1D), and 1 g/kg BM (Figure 1E) atherosclerotic characteristics were not observed. 

The mechanism of inhibitory capacity of EEP, as far as atherosclerosis is concerned, is difficult to explain. Our study has led to a conclusion that the mechanism is related to a significant decrease in total cholesterol, oxidatively modified pro-atherogenic ox-LDL molecules, and the decrease of ADMA and ANG II level. To the best of our knowledge, the current study is the first report on the subject.

EEP used in our study was characterized by a high content of polyphenols (27 mg GAE/g) and flavonoids (20 mg QE/g), which corresponded to a strong antioxidant effect resulting from reduction of DPPH (EC_50_ = 57.5 μmol/g), free radicals, and ABTS^•+^ (TEAC = 0.692 mmol/g). Rutin was a dominant flavonoid, which is a glycoside of quercetin. Other flavonoids present in the extract are: mireycetin > quercetin > isorhamnetin > kaempferol. The main phenolic acids which are present in EEP are: gallic acid > trans-cinnamic acid > 4-hydroxycinnaminic acid > felluric acid > 4-trans-p-coumaric acid > caffeic acid. Detailed characteristics of particular phenolic acid and flavonoid content in EEP used in research were presented in a previously published paper [22]. These compounds are characterized by a strong antioxidant effect reducing oxidative stress, and strongly inhibiting lipid peroxidation, which probably was crucial for preventing the formation of atherosclerotic plaque in ApoE-knockout mice.

In vitro studies showed that quercetin and catechin inhibit platelet aggregation, limit pro-clotting activity and block phosphoinositide cascade [24]. Flavonoids interact with platelet receptors. Quercetin decreases reactivity of platelets by blocking activation that depends on GPVI receptors [25]. Quercetin and catechin inhibit oxidative stress in platelets, and simultaneously limit activation of a fibrogen receptor, and increase NO synthesis [26]. In studies conducted on mice with apolipoprotein E deficiency, it was shown that polyphenol mixture, e.g., catechin, caffeic acid and resveratrol, decreases progression of atherosclerotic changes in the aortic arch. According to authors, the inhibition of endothelin 1 synthesis is one of the mechanisms in which polyphenols affect the balance between vasoconstrictive and vasodilating factors [27]. Hayek et al. [28] obtained significant reduction of atherosclerotic plaque on the surface of the aortic intima-media in ApoE-knockout mice after administration of quercetin.

The anti-atherosclerotic effect of propolis, an important apitherapeutic, was described in the literature. The reduction of early and advanced atherosclerotic changes due to a polyphenol fraction from green, brown, and red propolis was confirmed in in vitro studies conducted on genetically modified mice without LDL receptor (LDLr-/- mice). Polyphenols from red propolis had the strongest effect, and they reduced atherosclerotic changes in the aortic sinus. This was related to improving the lipid profile, reducing the level of pro-inflammatory cytokines, monocyte chemotactic protein-1 (MCP1) and interleukin 6 (IL6), chemokine, and angiogenic factors [29]. Limitation of atherosclerotic progression in the aortic sinus was observed in the case of a polyphenol fraction from Chilean propolis. Authors claim that this can result from the synergic effect of a polyphenol complex present in propolis that involves a significant reduction of the expression of a pro-angiogenic vascular endothelial growth factor A (VEGF-A) [30].

### 3.2. Effect of EEP on Total Cholesterol (TC)

Improper diet can impair lipid management and contribute to the development of cardio-vascular diseases. Disorders of lipid metabolism, manifested by hypercholesterolemia, are recognized and important factors which increase the risk of atherosclerosis [31].

**Figure 2:**
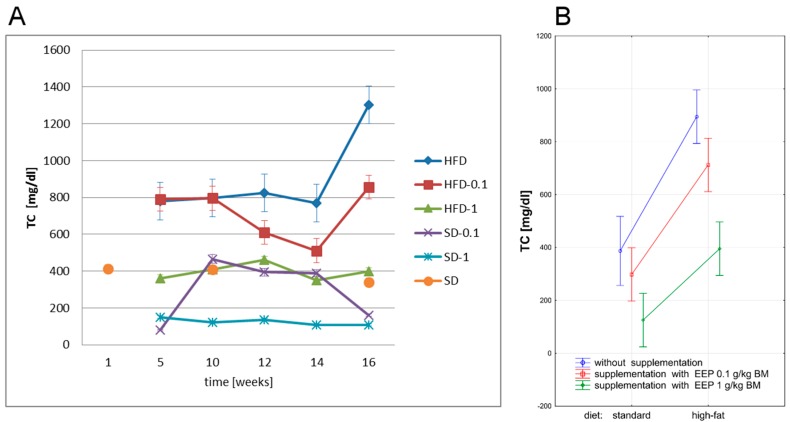
Total cholesterol (TC) level in ApoE-knockout mice. (**A**) TC level in particular weeks of the study; (**B**) Average TC level (*n* = 10). HFD—high-fat diet; HFD-0.1—high-fat diet supplemented with EEP 0.1 g/kg BM; HFD-1—high-fat diet supplemented with EEP 1 g/kg BM; SD-0.1—standard diet supplemented with EEP 0.1 g/kg BM; SD-1—standard diet supplemented with EEP 1 g/kg BM; SD—standard diet. In HFD-1 group, TC concentration during the total research period corresponded to TC level determined in SD. The lowest TC values were recorded for SD-1.

In the current study, we have evaluated the effect of polyphenol-rich extract of bee pollen (EEP) on total cholesterol level in ApoE-knockout mice on high-fat and standard diets. Total cholesterol level in ApoE-knockout mice is presented in Figure 2.

After five weeks, a high-fat diet in genetically modified animals resulted in an increase of TC to 779 mg/dL (Figure 2A), i.e., by 101% compared with the average level (388 mg/dL, Figure 2B) in the controls. The use of a high-fat diet for 16 weeks further increased the level of TC to 1303 mg/dL (Figure 2A), i.e., by 67% compared with values recorded after five weeks. In this group, the average TC level was the highest and the difference in comparison with all remaining experimental groups was statistically significant (*p* < 0.05, Figure 2B).

Supplementing a high-fat diet with EEP in a dose of 0.1 g/kg BM decreased TC level, which reached the value of 856 mg/dL (Figure 2A) in the 16th week, and was 34% lower when compared with an unsupplemented high-fat diet. Supplementation of a high-fat diet with EEP in a dose of 1 g/kg BM stabilized TC level as early as in the 5th week. This parameter level in all experimental periods was similar to the control group level, and the difference was not statistically significant (Figure 2B). In the 16th week, TC level was 398 md/dL (Figure 2A), i.e., 69% lower than in an unsupplemented high-fat diet.

Supplementation of a standard diet with EEP in a dose of 0.1 g/kg did not significantly change TC level (*p* > 0.05), but a dose of 1 g/kg MB resulted in a high decrease in TC level (*p* < 0.05) when compared with the control group (Figure 2A,B).

A significant decrease of TC level due to polyphenol fraction in bee pollen extract (EEP), which was recorded in our study, can be an effect of various mechanisms. It can be a sign of decreased biosynthesis of cholesterol in the liver. It can be a result of increased excretion of cholesterol with bile, or a result of the activation of peroxisome proliferator-activated receptors (PPAR), which regulate adipocyte maturation and lipid storage; therefore, they are regulators of hepatic metabolism of lipids.

It can be concluded from published data that polyphenols from natural products affect lipid metabolism as well as having cardio- and angio-protective effect. During in vitro studies, quercetin activates PPAR-γ receptors by increasing their expression. It also increases the expression of ATP-binding cassette transporter (ABCA1), which plays a key role in reverse cholesterol transport. Quercetin-induced modulation of ABCA1 expression reduces cholesterol build-up in macrophages, and increases cholesterol outflow from macrophages, and therefore reduces formation of foam cells, lowering the risk of atherosclerosis [32]. The lipid level in LDLr-knockout mice is normalized by a polyphenol fraction from different types of propolis. The polyphenol fraction from red propolis had the strongest protective effect. This fraction caused the most significant reduction of triglyceride level, and TC level as well as an increase in HDL. The anti-atherogenic effect was weaker in the case of green and brown propolis. This may be related to different content of main polyphenol components, in particular, types of propolis. The following were mostly present in red propolis: 3-hydroxy-8,9-dimethoxypterocarpan, medicarpin, daidzein, whereas artepellin C, pinocembrin, kampferol in green propolis, and pinocembrin, caffeic acid phenyl ester, quercetin, galangin in brown propolis [29].

The current study shows that EEP in a dose of 1 g/kg BM significantly decreases TC levels in ApoE-knockout mice on both, high-fat and standard diets. Consequently, it reduces hypercholesterolemia, which is a risk factor for atherosclerosis.

### 3.3. Effect of EEP on Oxidized Low Density Lipoprotein (Ox-LDL)

Oxidized LDL molecules are one of the main factors responsible for atherosclerosis development. They are formed due to free radical activity during the process of chemical modification, namely, oxidation of low density lipoproteins (LDL). Ox-LDL are highly atherogenic molecules, since they have pro-inflammatory activity and they cause focal inflammation of arterial vascular endothelium [33].

**Figure 3:**
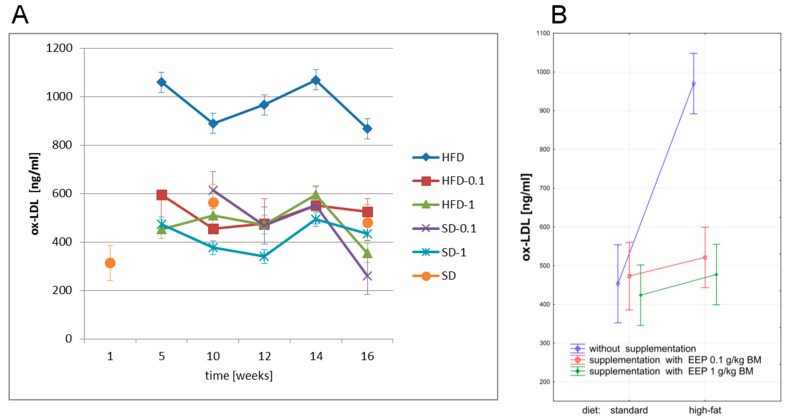
Oxidized low density lipoproteins (ox-LDL) level in ApoE-knockout mice. (**A**) Ox-LDL level in particular weeks of the study; (**B**) Average ox-LDL level (*n* = 10). HFD—high-fat diet; HFD-0.1—high-fat diet supplemented with EEP 0.1 g/kg BM; HFD-1—high-fat diet supplemented with EEP 1 g/kg BM; SD-0.1—standard diet supplemented with EEP 0.1 g/kg BM; SD-1—standard diet supplemented with EEP 1 g/kg BM; SD—standard diet. The highest values of ox-LDL were recorded in HFD group. In groups HFD-0.1 and HFD-1, the concentration of ox-LDL was much lower than in HFD.

In our study, we have investigated the effect of polyphenol-rich EEP on ox-LDL levels in ApoE-knockout mice on high-fat and standard diets. Ox-LDL level in ApoE-knockout mice is presented in Figure 3.

The highest levels of ox-LDL in all experimental periods were recorded in mice on a high-fat diet, whereas the lowest ones were noted when a standard diet was supplemented with EEP at a dose of 1 g/kg BM (Figure 3A,B). Since the fifth week, a high-fat diet resulted in a significant increase of ox-LDL level, which was 867 ng/mL in the 16th week (Figure 3A) and was 91% higher than the average level (453 ng/mL, Figure 3B) in the controls. Supplementing a high-fat diet with EEP as early as in the fifth week resulted in lowering ox-LDL levels, which diminished to 526 ng/mL (Figure 3A), i.e., by 40% in the case of a dose of 0.1 g/kg BM, and to 355 ng/mL (Figure 3A), i.e., by 59% when the dose was 1 g/kg BM compared with an unsupplemented high-fat diet. In Apo-E-knockout mice, supplementation of a high-fat diet with EEP regardless of the dose, causes statistically significant reduction of average ox-LDL level to a level similar to the one of the controls (Figure 3B).

The literature indicates that polyphenols are inhibitors of oxidative modification of LDL. They play an important role in prevention of many degenerative diseases, including cardio-vascular diseases. Polyphenols inhibit progression of atherosclerosis by reducing oxidative stress, and inflammatory biomarkers of atherosclerosis, as well as by improving lipid profile and insulin sensitivity, and by making endothelial function more efficient [17,34,35,36].

Loke et al. [37], in their studies on ApoE-knockout mice showed that quercetin reduced hydrogen peroxide and leukotrien B4 in vessels, and P-selectin serum level as well as increased the activity of endothelial nitric oxide synthase (eNOS). Theaflavin (catechin dimer) and epicatechin have a similar but weaker effect. In vitro studies of olive oil polyphenols have shown that they reduce intracellular levels of reactive oxygen species (ROS) and the expression of nuclear factor-κB (NFκB) transcription. Reduction of NFκB expression has been related to lowering metalloproteinase 9 level [36]. Oxidative stress in ApoE-knockout mice is also reduced by cocoa polyphenols. This effect has resulted from reducing expression of vascular cell adhesion molecule-1 (VCAM1) and intercellular adhesion molecule-1 (ICAM1) [38]. Olive oil supplemented with epigallocatechin gallate (EGCG) significantly improves vascular endothelium function in patients with early atherosclerotic dysfunction of endothelium due to the levels of ICAM, monocytes and lymphocytes [39]. Polyphenols inhibit LDL oxidation, reduce thrombocyte aggregation, inhibit the activity of enzymes that mediate the immune cells response to LDL, and reduce TC levels [40].

The presented effect of the polyphenol fraction of bee pollen extract on ox-LDL level in ApoE-knockout mice has been reported for the first time. Any information on the effect of polyphenols from bee pollen on ox-LDL level has not been found in the literature. Our study can lead to the conclusion that a polyphenol fraction from bee pollen significantly reduces ox-LDL level, and it results, most probably, from high antioxidant capacity of EEP. Reduction of proatherogenic ox-LDL level and consequently, limitation of the development of atherosclerotic changes are the signs that ethanol extract of bee pollen is highly efficient at reducing oxidative stress. Therefore, it can be useful as a potential anti-atherogenic substance.

### 3.4. Effect of EEP on Asymmetric Dimethylarginine (ADMA)

ADMA is an endogenous inhibitor of endothelial nitric oxide synthase (eNOS). ADMA participates in one of the mechanisms limiting bioavailability of nitric oxide (NO), which is an endogenous strongly anti-atherogenic substance [41,42]. Impairment of endothelial functions, which is observed in various disorders, results from high levels of ADMA. This allows us to single out ADMA as an early biochemical marker of endothelial dysfunction in the prophylaxis of cardio-vascular diseases and insulin resistance [42,43,44].

**Figure 4:**
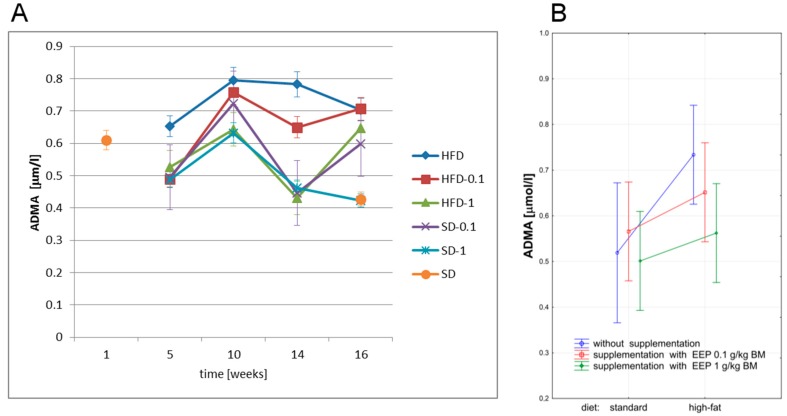
Asymmetric dimethylarginine (ADMA) level in ApoE-knockout mice. (**A**) ADMA level in particular weeks of the study; (**B**) Average ADMA level (*n* = 8). HFD—high-fat diet; HFD-0.1—high-fat diet supplemented with EEP 0.1 g/kg BM; HFD-1—high-fat diet supplemented with EEP 1 g/kg BM; SD-0.1—standard diet supplemented with EEP 0.1 g/kg BM; SD-1—standard diet supplemented with EEP 1 g/kg BM; SD—standard diet. In HFD-1 group, during the total period of observation, lower ADMA concentration than HFD concentration was recorded.

We have studied the effect of bee pollen extract on ADMA level in ApoE-knockout mice on both, high-fat and standard diets. ADMA level is presented in Figure 4. 

After five weeks, a standard diet led to an increases in ADMA level to 0.653 μmol/L (Figure 4A), i.e., by 26%, and up to 0.704 μmol/L (Figure 4A), i.e., by 36% after 16 weeks when compared with an average level (0.519 μmol/L, Figure 4B) in the controls.

Sixteen-week supplementation of a high-fat diet with EEP in a dose of 0.1 g/kg BM did not significantly change this parameter level. When a high-fat diet was supplemented with EEP in a dose of 1 g/kg BM, the lowest ADMA level was recorded in the 14th week (0.431 μmol/L, Figure 4A), while in the 16th week ADMA level was 0.648 μmol/L (Figure 4A), 8% lower than in the unsupplemented group. The lowest levels of this parameter were obtained for a standard diet supplemented with bee pollen extract in a dose of 1 g/kg BM (Figure 4A,B). A high-fat diet in ApoE-knockout mice caused a statistically significant increase of ADMA level. Supplementing a high-fat diet with EEP in a dose of 1 g/kg BM in ApoE-knockout mice resulted in a statistically significant decrease of average ADMA levels (Figure 4B).

According to the literature, polyphenols added to diet lower ADMA. Li Volti’s et al. [45] in vitro studies showed that supplementation with silibinin—flavonoid belonging to the group of flavonolignans—lowered ADMA serum level in mice, limited endothelial dysfunction, and reduced insulin resistance. A significant decrease of ADMA level, ox-LDL level and C-reactive protein (CRP) level, as a result of polyphenol-rich olive oil consumption, was observed in studies carried out in a group of young women with slight hypertension or hypertension stage 1. A significant decrease of hypertension and improving endothelial function was recorded [46].

There are no published reports on the effect of bee pollen extract on ADMA level. Our results show that a polyphenol fraction from bee pollen reduces ADMA level, and consequently increases NO bioavailability, by limiting endothelial dysfunction and development of atherosclerotic changes. It is very important for prevention of cardio-vascular diseases, because an increase of ADMA level is an early risk factor of vascular endothelium dysfunction, and limiting its excessive synthesis can be used in atherosclerotic prevention.

### 3.5. Effect of EEP on Angiotensin-Converting Enzyme (ACE) and Angiotensin II (ANG II)

The renin-angiotensin-aldosterone system (RAA) is a key mechanism of physiologic regulation of blood pressure and electrolyte balance. Individual constituents of the system can participate in pathogenesis of hypertension and organ changes related to hypertension such as cardiac and vascular remodeling, atherosclerosis, myocardial fibrosis, and renal fibrosis [47].

ACE belongs to zinc metalloproteinases. It is an enzyme that participates in blood pressure regulation through converting inactive angiotensin I into biologically active angiotensin II, with a vassopresor-like activity [48].

ANG II has a harmful effect on vascular endothelium and cardiac muscle. It causes endothelial dysfunction, increases oxidative stress, and contributes to atherosclerotic plaque rupture. ANG II also accelerates apoptosis of cardiac muscle cells, and increases tissue-specific insulin resistance [49]. By contributing to ox-LDL formation, ANG II can indirectly lead to dysfunctions of endothelial function related to clotting and fibrinolysis [50]. ANG II can also cause a change in endothelial function from anti-adhesive to pro-adhesive one, thus affecting the expression of endothelial adhesion molecules (ICAM, VCAM) [51].

**Figure 5:**
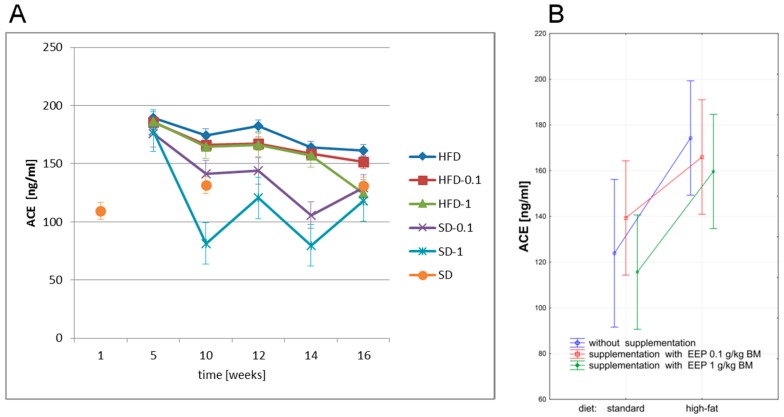
Angiotensin-converting enzyme (ACE) level in ApoE-knockout mice. (**A**) ACE level in particular weeks of the study; (**B**) Average ACE levels (*n* = 10). HFD—high-fat diet; HFD-0.1—high-fat diet supplemented with EEP 0.1 g/kg BM; HFD-1—high-fat diet supplemented with EEP 1 g/kg BM; SD-0.1—standard diet supplemented with EEP 0.1 g/kg BM; SD-1—standard diet supplemented with EEP 1 g/kg BM; SD—standard diet. In HFD-1, a decrease in ACE concentration was observed in the 16th week, when compared to HFD. The lowest levels of ACE were determined in SD-1.

In our study, we have investigated the effect of EEP on ACE and ANG II level in ApoE-knockout mice on high-fat and standard diets. ACE level is presented in Figure 5.

The highest levels of ACE in particular experimental periods were recorded in animals on a high-fat diet, whereas the lowest ones were observed for a standard diet supplemented with bee pollen extract in a dose of 1 g/kg BM (Figure 5A). After five weeks, a high-fat diet increased ACE level, which was 161 ng/mL (Figure 5A) in the 16th week, i.e., 30% higher than the average level (124 ng/mL, Figure 5B) in the control group. Supplementing a high-fat diet with EEP resulted in lowering this parameter level to 152 ng/mL after 16 weeks, i.e., by 6% for 0.1 g/kg BM, and to 124 ng/mL (Figure 5A) i.e., by 23% for a dose of 1 g/kg BM when compared with the unsupplemented group.

**Figure 6:**
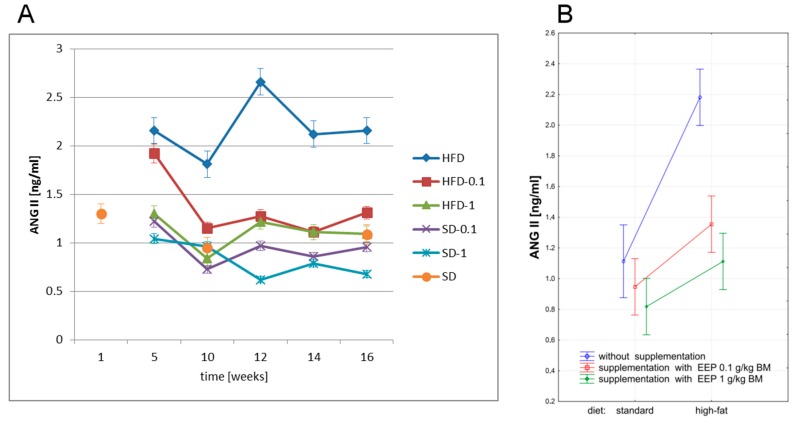
Angiotensin II (ANG II) level in ApoE-knockout mice. (**A**) ANG II level in particular week of the study; (**B**) Average ANG II level (*n* = 10). HFD—high-fat diet; HFD-0.1—high-fat diet supplemented with EEP 0.1 g/kg BM; HFD-1—high-fat diet supplemented with EEP 1 g/kg BM; SD-0.1—standard diet supplemented with EEP 0.1 g/kg BM; SD-1—standard diet supplemented with EEP 1 g/kg BM; SD—standard diet. The highest ANG II concentration was recorded in HFD. In HFD-0.1 and HFD-1, the concentration of ANG II during the whole research period was lower when compared to HFD.

The highest level of ANG II was recorded in mice on a high-fat diet. In the fifth and sixth week, ANG II level was 2.16 ng/mL (Figure 6A), i.e., 95% higher than the average level (1.11 ng/mL, Figure 6B) in the control group. Supplementing a high-fat diet with EEP decreased this parameter as early as in the fifth week, and after 16 weeks ANG II level was reduced to 1.31 ng/mL (Figure 6A), i.e., by 39% for a dose of 0.1 g/kg BM, and to 1.09 ng/mL (Figure 6A), i.e., by 50% for a dose of 1 g/kg BM compared with the unsupplemented group. The lowest ANG II level was recorded in mice on a standard diet supplemented with EEP in a dose of 1 g/kg BM (Figure 6A). Supplementation of a high-fat diet with bee pollen extract, regardless of the dose, caused a statistically significant decrease of ANG II level (Figure 6B).

The published data reveals that polyphenols are hypotensive, which results from their antioxidant capacity. This feature leads to lowering ROS concentration and to weakening ACE activity [19,51,52]. Xu et al. [53], both in studies on rats and in vitro studies showed that genistein had reduced expression and activity of ACE in endothelium and serum. The estrogen receptor activating a pathologic path of ERK1/2 participated in this activity. Genistein stimulates the synthesis of nitrogen oxide in endothelial cells through a mechanism dependent on cyclic 3′5′-adenosine monophosphate. Taxifolin inhibits ACE activity in the rat aorta [52], while quercetin lowers blood pressure in spontaneously hypertensive rats (SHR), and eliminates all pathological changes in their blood vessels [54]. The antihypertensive effect of polyphenols in SHRs was related to activation of eNOS, and inhibition of metalloproteinase-2 [55]. Lowering blood pressure by polyphenols can also result from modelling the renin-angiotensin-aldosterone system through reducing oxidative stress [56]. Polyphenols from various tea types inhibit ACE activity in in vivo studies [57]. Furthermore, also bee pollen extract has inhibited ACE activity in in vitro studies, which results from its high antioxidant potential [58].

Based on our study, it can be concluded that supplementing a high-fat diet and a standard diet with EEP decreases angiotensin II level in ApoE-knockout mice. This is related to weakening the activity of ACE. The available literature does not offer any data on the effect of bee pollen on ANG II level; this issue has been presented in our study for the first time. Our study may lead to a conclusion that a polyphenol fraction from bee pollen, due to its high antioxidant capacity, affects the modulation of the renin-angiotensin-aldosterone system; therefore, it improves endothelial function, and consequently it can inhibit the development of atherosclerotic changes.

In spite of the fact that many studies have been conducted, the mechanism of the anti-atherosclerotic effect has not been yet determined. According to the published data, it can be assumed that the main protective effect from the development of atherosclerosis consists improvement of endothelial function, oxidative reduction, LDL modification, decrease of total cholesterol, inhibiting the synthesis of pro-inflammatory cytokines such as TNF-α, IL-6, IL-8, and inhibiting the adhesion of ICAM-1, VCAM-1 molecules as well as the stimulation of eNOS synthase, and intensification of NO synthase [59].

## 4. Conclusions

Supplementing a high-fat diet with polyphenol-rich EEP modulates lipid profile in ApoE-knockout mice by lowering total cholesterol level. EEP is protective for cardiac arteries since it significantly limits the development of atherosclerotic changes (dose of 0.1 g/kg BM) or prevents their development (dose of 1 g/kg BM). This results from lowering the level of strongly pro-atherosclerotic ox-LDL. Due to high antioxidant activity, EEP efficiently reduces oxidative stress, and is hence an inhibitor of LDL oxidation. The protective effect of EEP results from lowering ADMA level, which increases NO bioavailability, limits endothelial dysfunction, and prevents the development of atherosclerotic changes. An increase in ADMA level is an early risk factor of endothelium dysfunction, and limiting its excessive synthesis is crucial and can be used in prophylaxis of atherosclerosis as well as other cardio-vascular diseases. EEP prevents and inhibits the development of atherosclerotic changes in ApoE-knockout mice through inhibiting the activity of ACE and lowering ANG II level. Consequently, it affects modulation of the renin-angiotensin-aldosterone system, and can limit endothelial dysfunction in this way. Further studies should aim at comprehensive explanation of both, cellular and genetic mechanisms of a cardioprotective effect of polyphenols, including their stability, absorption, distribution, and biotransformation. 

To the best of our knowledge, we have been the first to prove that in experimental conditions, EEP efficiently reduces oxidative stress through limiting atherosclerotic changes or eliminating their development. Hence, EEP can be useful as a potential anti-atherogenic agent.
